# Assessing the influence of preconception diet on female fertility: a systematic scoping review of observational studies

**DOI:** 10.1093/humupd/dmad018

**Published:** 2023-07-19

**Authors:** Simon Alesi, Nahal Habibi, Thais Rasia Silva, Nicole Cheung, Sophia Torkel, Chau Thien Tay, Alejandra Quinteros, Hugo Winter, Helena Teede, Aya Mousa, Jessica A Grieger, Lisa J Moran

**Affiliations:** Monash University, Clayton, VIC, Australia; Adelaide Medical School, The University of Adelaide, Adelaide, SA, Australia; Robinson Research Institute, The University of Adelaide, Adelaide, SA, Australia; Postgraduate Program in Endocrinology and Metabolism, Universidade Federal do Rio Grande do Sul, Porto Alegre, Brazil; Adelaide Medical School, The University of Adelaide, Adelaide, SA, Australia; Monash University, Clayton, VIC, Australia; Monash University, Clayton, VIC, Australia; Adelaide Medical School, The University of Adelaide, Adelaide, SA, Australia; Monash University, Clayton, VIC, Australia; Monash University, Clayton, VIC, Australia; Monash University, Clayton, VIC, Australia; Adelaide Medical School, The University of Adelaide, Adelaide, SA, Australia; Robinson Research Institute, The University of Adelaide, Adelaide, SA, Australia; Monash University, Clayton, VIC, Australia; Robinson Research Institute, The University of Adelaide, Adelaide, SA, Australia

**Keywords:** preconception diet, female infertility, Mediterranean diet, macronutrients, ART

## Abstract

**BACKGROUND:**

Preconception diet is a proposed modifiable risk factor for infertility. However, there is no official guidance for women in the preconception period as to which dietary approaches may improve fertility.

**OBJECTIVE AND RATIONALE:**

A comprehensive synthesis of the relevant evidence is key to determine the potentially effective dietary patterns and components as well as evidence gaps, and to provide information for nutritional recommendations for couples planning a pregnancy.

**SEARCH METHODS:**

In this systematic scoping review, four electronic databases (Medline and EMBASE via Ovid processing, CAB Direct, and CINAHL via EBSCO) were searched for observational studies (prospective and retrospective cohort, cross-sectional, and case–control studies) from inception to 27 September 2021. Eligible studies included women of reproductive age during the preconception period, and evaluated exposures related to preconception diet and outcomes related to fertility. Results were synthesized using a descriptive approach.

**OUTCOMES:**

A total of 36 studies were eligible for inclusion (31 prospective, 3 cross-sectional, and 2 case–control studies) and were published between 2007 and 2022. Of the assessed dietary exposures, increased adherence to the Mediterranean diet displayed the strongest and most consistent association with improved clinical pregnancy rates. Reducing trans fatty acids (TFAs), saturated fatty acids, and discretionary food intake (fast food and sugar-sweetened beverages) were associated with improvements in live birth, clinical pregnancy rates, and related ART outcomes. The dietary components of seafood, dairy, and soy demonstrated inconsistent findings across the few included studies.

**WIDER IMPLICATIONS:**

Due to heterogeneity and the limited available literature on most exposures, there is insufficient evidence to support any specific dietary approach for improving fertility. However, following some of the dietary approaches outlined in this review (anti-inflammatory diets, reducing TFA, and discretionary food intake) are consistent with broad healthy eating guidelines, have little to no associated risk, and offer a plausible set of possible benefits. This warrants further exploration in randomized controlled trials.

## Graphical Abstract

**Figure:**
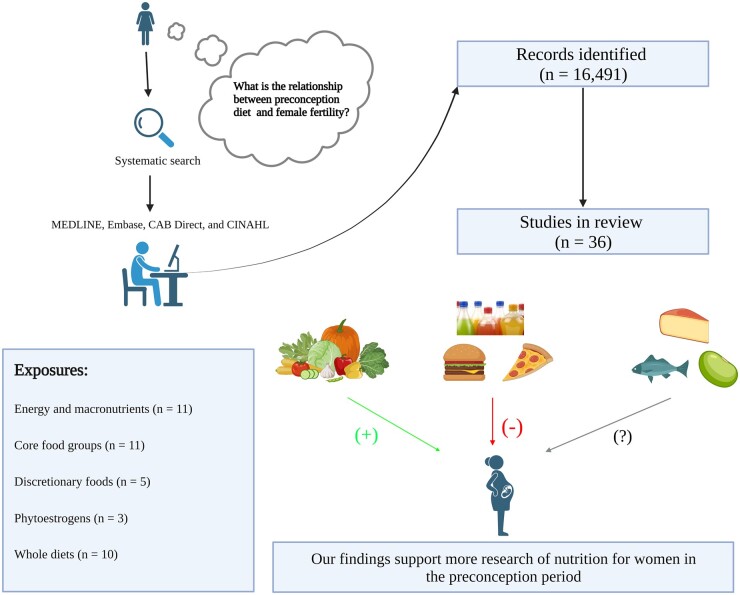
Systematic search for preconception nutrition in female fertility identified that the Mediterranean diet and reducing discretionary foods/drinks yielded benefits; however, there was limited evidence for some foods.

## Introduction

Infertility is defined as the failure to successfully conceive after more than 1 year of unprotected intercourse (Tabong and Adongo, 2013). Infertility affects 48 million couples and 186 million individuals globally (Word Health Organisation, 2022). Around 50% of all cases of infertility are due to female-factor infertility while 20–30% are due to a combination of both male and female factor infertility (Agarwal *et al.*, 2015). Infertility places a heavy burden on couples who wish to conceive, creating negative psychological sequelae including anxiety, depression, and stress (Yusuf, 2016). This is further coupled with significant physical and economic challenges. Moreover, in spite of recent advances in ART, the cost of infertility remains high, with one IVF cycle costing upwards of US$12 513, US$5244, and US$5645 in the USA, UK, and Australia, respectively (Teoh and Maheshwari, 2014). Therefore, there is a need for relatively simple and inexpensive modifiable risk factors to be explored as potentially new or adjunct avenues to fertility treatment.

Modifiable lifestyle-related risk factors, such as suboptimal preconception nutrition, obesity, anxiety, and stress, are consistently associated with a higher likelihood of infertility and poor fertility outcomes (Dağ and Dilbaz, 2015; Panth *et al.*, 2018; Rooney and Domar, 2018). Preconception diet is a key modifiable risk factor for infertility, with many women having inadequate nutritional intake in the preconception period (Awoke *et al.*, 2022). This has generated considerable interest around the relevance of diet to reproductive health. Several cross-sectional and prospective studies have indicated that modifying preconception dietary patterns to align with international food-based dietary guidelines may be beneficial for fertility and reproductive outcomes such as ovulation and menstrual regularity in a variety of geographical populations (Chavarro *et al.*, 2007a; Herforth *et al.*, 2019; Krabbenborg *et al.*, 2021). Limiting intake of discretionary foods which include higher amounts of trans (TFAs) and saturated fatty acids (SFAs), sodium and free sugars, while promoting intake of unsaturated fats and core foods such as whole grains, dairy, vegetables, and fish, may improve reproductive success (Herforth *et al.*, 2019). Whilst the exact mechanism in which these core food components improve fertility are largely unknown, the increased inflammation and oxidative stress from higher intakes of discretionary choices are thought to play a key role for poorer fertility outcomes (Ley *et al.*, 2014; Koebnick *et al.*, 2018; Lin *et al.*, 2020; Wu *et al.*, 2020; Mazidi *et al.*, 2021).

Despite growing acceptance that diet is associated with reproductive outcomes in women, there remains no official guidance for women in the preconception period regarding which dietary (or duration) approaches to follow for optimal fertility. Synthesizing the relevant evidence and identifying key dietary patterns and components is pertinent to developing knowledge in this field and providing an evidence base to assist with formulating evidence-based nutritional recommendations for couples planning a pregnancy. Due to the lack of randomized controlled trials (RCTs) assessing specific dietary components or patterns in nutrition research for the purposes of improving fertility outcomes, using existing observational evidence can aid in identifying targets to assess in future intervention studies (Ioannidis, 2016). Therefore, the purpose of this scoping review is to examine the extent and range of observational research undertaken to evaluate the effect(s) of preconception dietary intakes and patterns on fertility outcomes.

## Methods

### Research question

The research question for this review is: What is the relationship between preconception diet and female fertility?

To address this research question, we focussed on observational studies that assessed dietary approaches among women attempting pregnancy and/or collected diet data prior to pregnancy.

### Eligibility criteria

Eligibility was determined using the Participant-Exposure-Comparison-Outcome (PECO) framework, defined a priori in the protocol, which is registered on the Open Science Framework (OSF) database (10.17605/OSF.IO/FBV6W).

#### Participants (P)

Participants included women of reproductive age, specifically during pre-conception (or inter-conception), pregnancy, and post-partum periods. Preconception was defined as the time when planning a pregnancy, and inter-conception was defined as the time in-between pregnancies. Pregnant and post-partum women were included when preconception behaviours were retrospectively assessed.

#### Exposure (E)

Dietary or nutritional components (i.e. whole diets, dietary patterns, food groups, or individual foods) were included as relevant exposures. Trials based solely on micronutrients, caffeine or alcohol, and studies with the stated goal of weight loss were excluded.

#### Comparison (C)

We included studies both with or without comparator groups.

#### Outcomes (O)

Studies were included if they reported any of the following outcomes: anovulatory or ovulatory fertility, conception via ART, pregnancy rate (clinical or biochemical), live birth rate, time to conception (natural or via ART), fecundity, ovulation, menstrual regularity, ART outcomes (e.g. fertilization rate, implantation rate, number of oocytes retrieved, number of cycles), stillbirth, miscarriage, or adverse ART outcomes (e.g. ovarian hyperstimulation syndrome, early pregnancy loss, multiple pregnancies).

### Study selection

#### Search strategy

The search strategy, including database selection and search terms (MeSH headings and keywords), was developed via consultation with experts in fertility or nutrition or scoping review methodology, and an expert medical librarian. A variety of keywords, relating to preconception, diet, and fertility, were used in the search strategy (Supplementary File S1). The following databases were searched: MEDLINE (Ovid), EMBASE (Ovid), CAB Direct, and CINAHL Plus (EBSCO). All sources were searched from inception to 27 September 2021. We also included additional studies based on expert opinion that were not identified in the original search. No studies were excluded due to being in a language other than English.

#### Screening process

Screening was undertaken using Covidence (www.covidence.org). Title and abstract screening were assessed in duplicate by several reviewers (S.A., N.H., T.R.S., N.C., A.Q., H.W., S.T., and J.G.), and full text screening was conducted (S.A., N.H., T.R.S., A.M., C.T.T., H.W., S.T., J.G., and L.M.) with 10% of articles being assessed in duplicate.

#### Data extraction and synthesis

Data were extracted (S.A., N.H., T.R.S., C.T.T., H.W., and S.T.) with 10% duplicate extraction (L.M. and J.G.) and a further 10% cross-checking to ensure the accuracy of the data extraction measures. A Microsoft excel spreadsheet was developed and pilot tested for data collection, with the final items being extracted: study details (author, year of publication, country of origin, study design, population and sample size, exposure/s, diet intake measurement, and duration of study), participants (population and setting), and outcomes (live birth, clinical pregnancy, early pregnancy loss, ovulatory infertility, miscarriage or stillbirth, time to pregnancy, fecundity, and IVF outcomes). As the role of scoping reviews is to represent the scope or coverage of a body of literature over time, it was not required to assess the study quality of the literature (Munn *et al.*, 2018).

## Results

**Figure 1.**
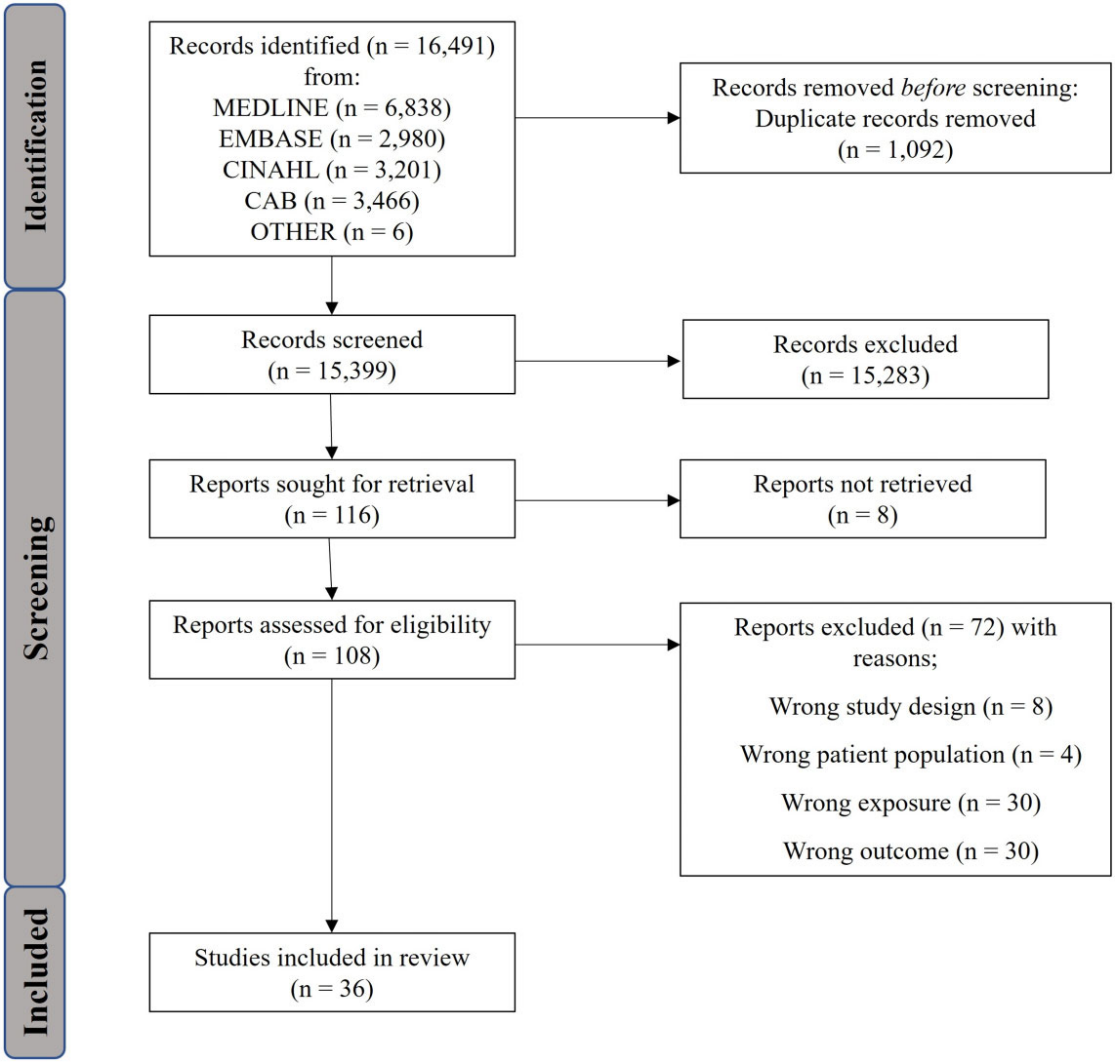
**PRISMA diagram for literature search process**.

Of the 16 491 articles identified, 1092 were removed as duplicates, leaving 15 399 studies for screening. After initial screening, there were 108 full texts assessed for eligibility, with eight records not being retrieved. This screening resulted in 36 studies being included in the scoping review (Fig. 1). Most of the included studies were prospective by design (Chavarro *et al.*, 2007a,b,c, 2008, 2009; Vujkovic *et al.*, 2010; Hatch *et al.*, 2012, 2018; Gaskins *et al.*, 2014, 2016, 2018, 2019; Jacobsen *et al.*, 2014; Vanegas *et al.*, 2015; Afeiche *et al.*, 2016; Machtinger *et al.*, 2017; Wise *et al.*, 2017, 2018, 2020; Chiu *et al.*, 2018b; Grieger *et al.*, 2018; Karayiannis *et al.*, 2018; Nassan *et al.*, 2018; Jahangirifar *et al.*, 2019; Ricci *et al.*, 2019; Sun *et al.*, 2019; Noli *et al.*, 2020; Wesselink *et al.*, 2020; Willis *et al.*, 2020; Hartman *et al.*, 2021; Salas-Huetos *et al.*, 2022), while three were cross-sectional (Revonta *et al.*, 2010; Lee *et al.*, 2020; Diba-Bagtash *et al.*, 2021), and two were nested case–control (Toledo *et al.*, 2011; Qu *et al.*, 2019) studies; their characteristics and results are outlined in Supplementary Tables S1 and S2.

**Figure 2.**
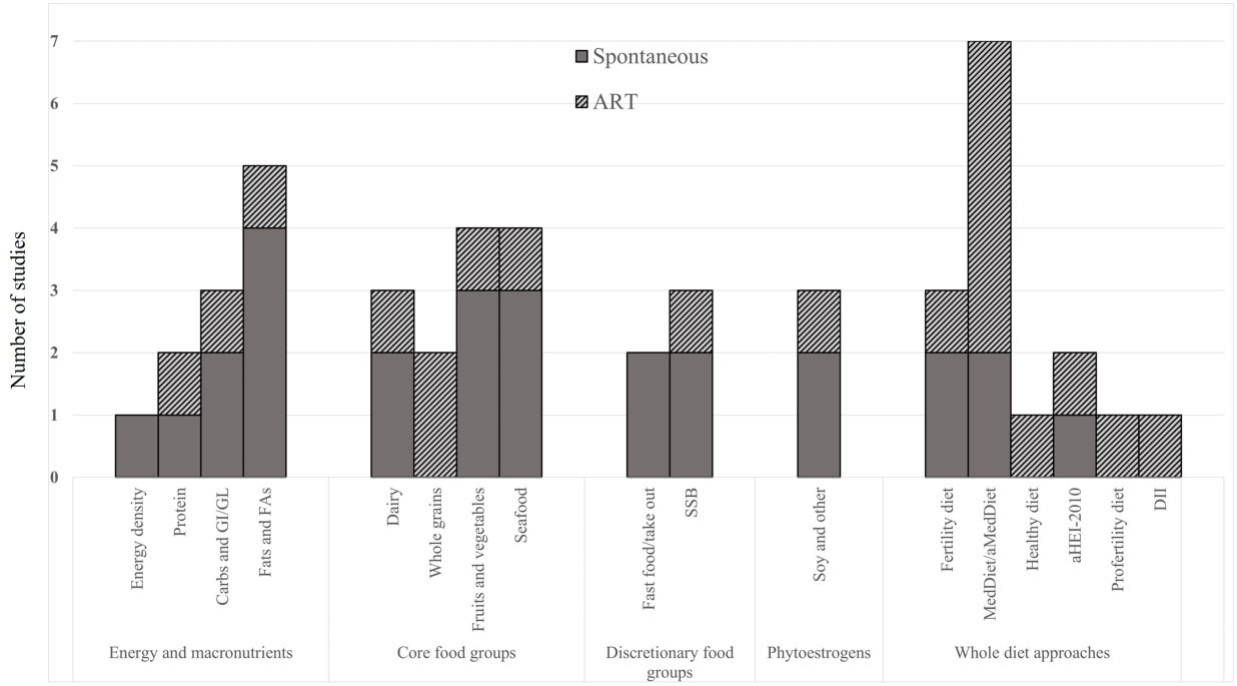
**The number of included studies regarding ART or spontaneous conception with the various primary or secondary exposures related to the main categories of diet-related categories.** aHEI-2010, alternative healthy eating index 2010; aMedDiet, alternative Mediterranean diet; Carbs, carbohydrates; ART, assisted reproductive technologies; DII, dietary inflammatory index; FAs, fatty acids; GI/GL, glycemic index/glycemic load; MedDiet, Mediterranean diet; SSB, Sugar-sweetened beverages.

Figure 2 details the number of studies relevant to energy and macronutrients, core food groups, discretionary food groups, phytoestrogens, or whole diet approaches, split by populations using or not using ART. Eleven studies included energy and macronutrients, such as energy density (n = 1), protein (n = 2), carbohydrates and glycemic index/load (n = 3), and total fat and fatty acid intake (n = 5). Twelve studies included core food groups, i.e. dairy (n = 3), whole grains (n = 2), fruits and/or vegetables (n = 4), and fish/seafood intake (n = 4). Five studies included discretionary foods, such as fast food and/or non-home prepared meals (n = 2) and sugar-sweetened beverages (n = 3). Three studies included phytoestrogens. Several exposures utilizing a whole of diet approach were also included; i.e. adherence to the fertility dietary pattern (n = 3), a Mediterranean diet (MedDiet/alternative MedDiet) (n = 7), a Healthy Eating Index-2010 (n = 2), a ‘pro-fertility diet’ (n = 1), a ‘healthy diet’ (n = 1), and the dietary inflammatory index (n = 1).

**Figure 3.**
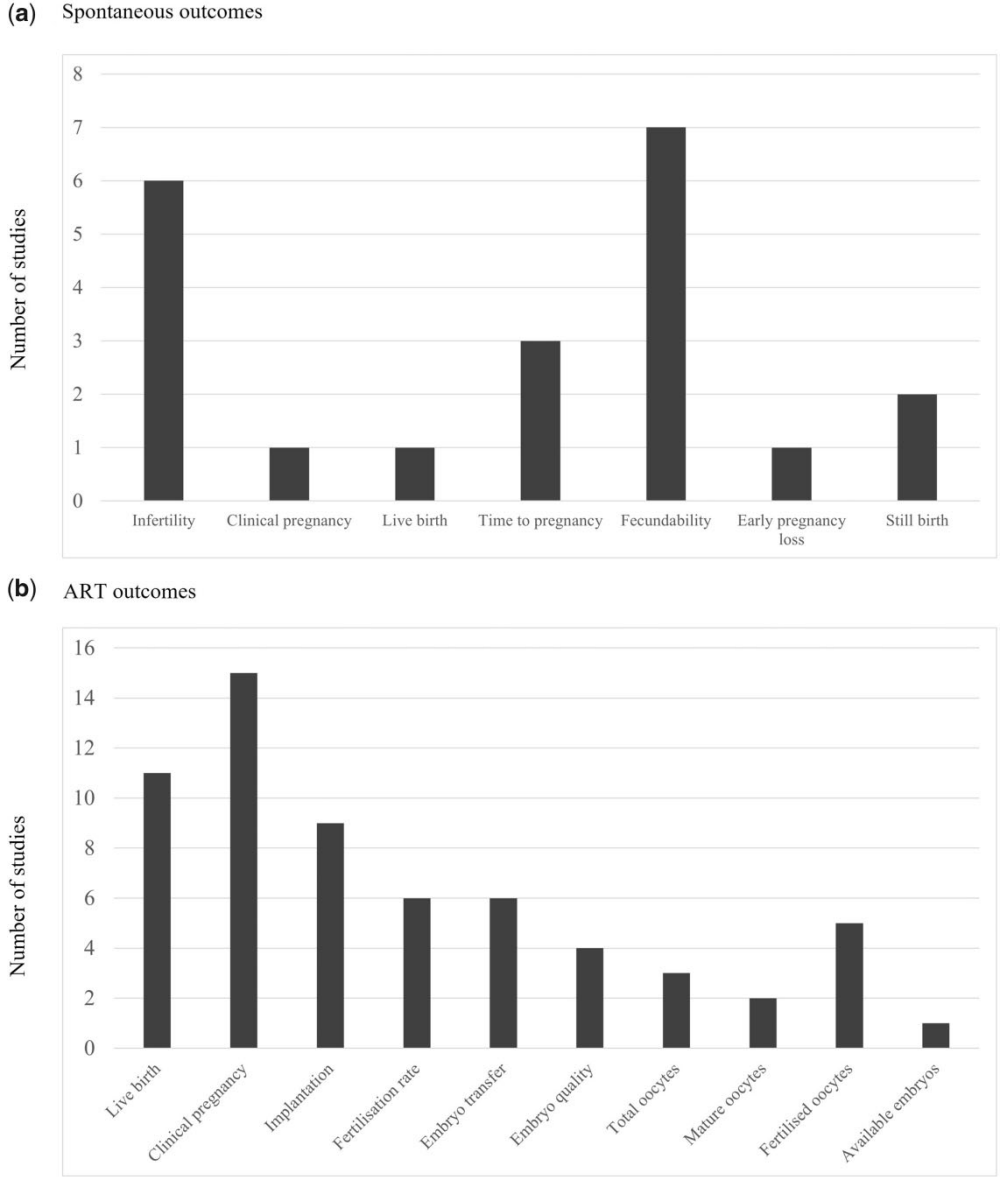
**The number of included studies for the reported outcomes among the populations.** (**A**) women conceiving spontaneously and (**B**) women conceiving with ART.

Figure 3 stratifies the number of outcomes for both spontaneous and ART outcomes in the included studies. The spontaneous fertility outcomes included infertility (n = 6), clinical pregnancy (n = 1), live birth (n = 1), time to pregnancy (n = 3), fecundability (n = 7), early pregnancy loss (n = 1), and stillbirth (n = 2). The ART outcomes included live birth (n = 11), clinical pregnancy (n = 15), implantation (n = 9), fertilization rate (n = 6), and IVF-specific outcomes such as embryo transfer (n = 6), embryo quality (n = 4), total oocytes retrieved (n = 3), mature oocytes retrieved (n = 2), number of fertilized oocytes (n = 5), and available embryos (n = 1).

### Energy and macronutrients

#### Energy density

One prospective cohort study of 132 spontaneously reproducing women examined energy density (Hartman *et al.*, 2021). Higher energy density was associated with a reduced clinical pregnancy rate (>1.6 kcal/g vs 1.37–1.6, Odds ratio (OR) (95% CI) 0.30 (0.11, 0.81)), reduced fecundity (indicated by increased time to pregnancy) (1.60 kcal/g vs 1.37–1.60, Hazard ratio (HR) (95% CI): 0.40 (0.21, 0.82)), and a reduced live birth rate (T3 vs T2, OR (95% CI): 0.43 (0.16, 1.07)), when modelled as a categorical variable but not when modelled as a continuous variable (Table 1).

**Table 1:** Results for fertility outcomes stratified by nutritional component in spontaneously conceiving women.

Category	Studies	Fertility	Clinical pregnancy	Live birth	Time to pregnancy	Fecundability	Early pregnancy loss and stillbirth
**ENERGY AND MACRONUTRIENTS**							

Energy density	1	–	NS: continuous. −ve: categorical >1.6 vs 1.37–1.6 (Hartman *et al.*, 2021).	−ve: third vs second energy density tertile (Hartman *et al.*, 2021).	NS: continuous +ve: categorical >1.6 vs 1.36–1.6 kcal/g (Hartman *et al.*, 2021).	–	–
Protein	1	−ve: increasing animal protein (Chavarro *et al.*, 2008). +ve: increasing vegetable protein (Chavarro *et al.*, 2008).	–	–	–	–	–
CHO	2	−ve: increasing % CHO (Chavarro *et al.*, 2009).	–	–	–	−ve: increasing added sugar (g/day) (Willis *et al.*, 2020). −ve: categorical increasing CHO-fiber ratio (Willis *et al.*, 2020).	–
GI/GL	2	−ve: increasing GI only in nulliparous (Chavarro *et al.*, 2009). –ve: increasing GL (Chavarro *et al.*, 2009).	NS: GI and GL (Noli *et al.*, 2020).	–	–	+ve: reduced categorical GL >141 vs >100 (Willis *et al.*, 2020).	–
Fats/FAs	4	NS: total fat, cholesterol, most FAs (Chavarro *et al*., 2007c). −ve: increasing TFAs (total, in place of n-6 PUFAs) (Chavarro *et al*., 2007c). −ve: higher PUFAs, higher TFA in infertile women <50 years (Revonta *et al.*, 2010).	–	–	–	−ve: increasing TFA (Wise *et al.*, 2018). +ve: increasing omega-3 (Wise *et al.*, 2018). NS: marine-sourced long chain fatty acids (Wise *et al.*, 2020).	–

**CORE FOOD GROUPS**							

Dairy	2	−ve: increasing low fat dairy (serving/week) ( Chavarro *et al*., 2007a). +ve: increasing high fat dairy foods (Chavarro *et al*., 2007a). +ve: increasing dairy fat (Chavarro *et al*., 2007a).	–	–	–	+ve: increasing dairy NS: low, high-fat dairy (Wise *et al.*, 2017).	–
Fruits and vegetables	3	+ve: increasing fruit (Grieger *et al.*, 2018). NS: fruits, vegetables (Revonta *et al.*, 2010).	–	–	+ve: increasing fruit. NS: vegetable (Grieger *et al.*, 2018).	–	+ve: low appetite for vegetables (Qu *et al.*, 2019).
Fish/seafood	3	NS: fish (Grieger *et al.*, 2018).	–	–	NS: fish (Grieger *et al.*, 2018).	NS: total seafood, unfried shellfish. −ve: Increasing fried shellfish (Wise *et al.*, 2020). +ve: Increased seafood servings/cycle (Gaskins *et al.*, 2018).	–
**DISCRETIONARY FOODS**							

Fast food/take-out	2	−ve: increasing fast food (Lee *et al.*, 2020). –ve: increasing non-home prepared meals/day.	–	–	−ve: increasing fast food (Grieger *et al.*, 2018).	−ve: Increasing non-home prepared meals/day.	–
Sugar-sweetened beverages	2	–	–	−ve: increasing SSB- fewer retrieved, mature, fertilized oocytes. NS: diet soda (Machtinger *et al.*, 2017).	–	−ve: increasing SSB, soda. NS: sugar-sweetened fruit juice and energy drinks (Hatch *et al.*, 2012). −ve: increasing SSB, soda (Hatch *et al.*, 2018).	–

**PHYTOESTROGENS**							

Soy and other	2	–	–	−ve: increasing isoflavone intake (g/day) (Jacobsen *et al.*, 2014). NS: parity: isoflavone intake (g/day) (Jacobsen *et al.*, 2014). −ve: nulligravidity higher with increasing isoflavone intake (g/day) (Jacobsen *et al.*, 2014).	–	NS: phytoestrogen (Wesselink *et al.*, 2020).	–

**WHOLE DIETS**							

Fertility diet	2	+ve: increasing dietary adherence (Chavarro *et al*., 2007b).	–	–	–	–	NS: early pregnancy loss, spontaneous abortion, and stillbirth (Gaskins *et al.*, 2014).
MedDiet/aMedDiet	2	–	+ve: increasing dietary adherence (Toledo *et al.*, 2011).	–	–	–	NS: early pregnancy loss, spontaneous abortion, and stillbirth (Gaskins *et al.*, 2014).
aHEI-2010	1	–	–	–	–	–	NS: early pregnancy loss, spontaneous abortion, and stillbirth (Gaskins *et al.*, 2014).

aHEI-2010, alternative healthy eating index 2010; aMED, alternative Mediterranean diet; CHO, carbohydrates; FAs, fatty acids; GI/GL, glycemic index/glycemic load; MedDiet, Mediterranean diet; NS, not significant; −ve/+ve, negative/positive; PUFA, polyunsaturated fatty acid; SSB, sugar sweetened beverages; TFA, trans saturated fatty acid.

#### Protein

Two prospective cohort studies assessed protein intake, one focussing on either the source of protein (vegetable or animal source) in 18 555 spontaneously reproducing women (Chavarro *et al.*, 2008), and one focussing on protein-rich foods in 351 women undergoing ART (Nassan *et al.*, 2018). Replacing 5% of animal protein with vegetable protein was associated with a 50% reduced risk of ovulatory infertility (Chavarro *et al.*, 2008). However, Nassan *et al.* (2018) reported no association between vegetable sources of protein (beans, nuts, and soy) and ART outcomes, including implantation, clinical pregnancy, and live birth (Table 2). Whilst consumption of protein-rich foods such as total meat intake and eggs was not associated with any reported ART outcome, increasing fish intake by two servings/week was positively related to live birth rate as well as a range of successful ART outcomes (clinical pregnancy, implantation, and others) (Nassan *et al.*, 2018) (Table 2). Unprocessed red meat intake (beef, pork, or lamb as the main meal), assessed as a continuous variable, was not associated with live birth rates. However, when comparing the lowest category of intake (no unprocessed red meat intake) compared to 0.36 servings/day, there was an associated 16.6% higher live birth rate (Nassan *et al.*, 2018) (Table 2).

**Table 2:** Results for fertility outcomes stratified by nutritional component in women conceiving via ART.

Category	Studies	Live birth	Clinical pregnancy	Implantation	Fertilization rate	IVF-specific (embryo and oocyte characteristics)
**ENERGY AND MACRONUTRIENTS**						

Protein	1	+ve: increasing quartiles of fish, also when replacing other meat, other protein-rich food (Nassan *et al.*, 2018). NS: total meat intake, eggs, vegetable source of protein (Nassan *et al.*, 2018).	NS: total meat intake, eggs, vegetable source of protein (Nassan *et al.*, 2018).	NS: total meat intake, eggs, vegetable source of protein (Nassan *et al.*, 2018).	–	–
CHO	1	NS: total CHO (Noli *et al.*, 2020).	NS: total CHO Noli *et al.* (2020).	–	–	NS: embryo transfer: total CHO (Noli *et al.*, 2020).
GI/GL	1	NS: Noli *et al.* (2020).	NS: Noli *et al.* (2020).	–	–	NS: embryo transfer (Noli *et al.*, 2020).
Fats and FAs	1	+ve: increasing DHA + EPA consumption (Salas-Huetos *et al.*, 2022). NS: total omega-3 consumption (Salas-Huetos *et al.*, 2022).	NS: all fats and fatty acids (Salas-Huetos *et al.*, 2022).	NS: all fats and fatty acids (Salas-Huetos *et al.*, 2022).	–	–

**CORE FOOD GROUPS**						

Dairy	1	+ve: increasing calorie and age-adjusted dairy intake in women >35 years old (Afeiche *et al.*, 2016). NS: full-fat or low-fat dairy (Afeiche *et al.*, 2016).	–	–	–	–
Whole grains	2	+ve: increasing whole grain intake (g/day) (Gaskins *et al.*, 2016). NS: Noli *et al.* (2020).	NS: Noli *et al.* (2020).	–	–	+ve: Endometrial thickness: Increasing whole grain intake (g/day) (Gaskins *et al.*, 2016). NS: Embryo transfer (Noli *et al.*, 2020).
Fish/seafood	1	+ve: Q1 vs Q4 total fish consumption (Salas-Huetos *et al.*, 2022).	NS: total fish consumption (Salas-Huetos *et al.*, 2022).	NS: total fish consumption (Salas-Huetos *et al.*, 2022).	–	–
Fruits and vegetables	1	Total fruits and vegetables: NS (Chiu *et al.*, 2018b)	Total fruits and vegetables: NS (Chiu *et al.*, 2018b)	Total fruits and vegetables: NS (Chiu *et al.*, 2018b).	–	–

**DISCRETIONARY FOODS**						

Sugar-sweetened beverages	1	+ve: Reducing cups of sugared soda/day (Machtinger *et al.*, 2017).	–	–	–	−ve: oocytes retrieved, mature oocytes retrieved, fertilized oocytes: SSB consumption compared to none (Machtinger *et al.*, 2017). NS: diet soda (Machtinger *et al.*, 2017).

**PHYTOESTROGENS**						

Soy and other	1	+ve: any soy isoflavone intake vs none (Vanegas *et al.*, 2015).	+ve: any soy isoflavone intake vs none (Vanegas *et al.*, 2015).	NS: soy isoflavone intake (Vanegas *et al.*, 2015).	+ve: any soy isoflavone intake vs none (Vanegas *et al.*, 2015).	–
**WHOLE DIETS**						

Fertility diet	1	NS (Gaskins *et al.*, 2019).	NS (Gaskins *et al.*, 2019).	NS (Gaskins *et al.*, 2019).	–	–
MedDiet/aMedDiet	5	+ve: increasing dietary adherence (Gaskins *et al.*, 2019). +ve: increasing dietary adherence in women <35 years old (Karayiannis *et al.*, 2018).	+ve: increasing dietary adherence in women >35 years old (Ricci *et al.*, 2019). +ve: increasing dietary adherence (Vujkovic *et al.*, 2010). NS (Sun *et al.*, 2019) +ve: increasing dietary adherence in women <35 years old (Karayiannis *et al.*, 2018).	NS (Sun *et al.*, 2019). NS (Karayiannis *et al.*, 2018).	NS (Vujkovic *et al.*, 2010).	−/+ve: viable embryos: increasing dietary adherence (Vujkovic *et al.*, 2010). +ve: fertilized oocytes and embryo yield: increasing dietary adherence (Sun *et al.*, 2019). NS: embryo quality (Vujkovic *et al.*, 2010). NS: number of good-quality embryos, good-quality oocytes, embryo transfer (Ricci *et al.*, 2019).
Profertility diet	1	+ve: increasing dietary adherence (Gaskins *et al.*, 2019).	+ve: increasing dietary adherence (Gaskins *et al.*, 2019).	+ve: increasing dietary adherence (Gaskins *et al.*, 2019).	–	–
aHEI-2010	1	NS (Gaskins *et al.*, 2019).	NS (Gaskins *et al.*, 2019).	NS (Gaskins *et al.*, 2019).	–	–
Healthy diet/unhealthy diet	1	–	−ve: T2 vs T1 ‘unhealthy diet’ adherence (Jahangirifar *et al.*, 2019). NS (Jahangirifar *et al.*, 2019).	–	NS (Jahangirifar *et al.*, 2019).	+ve: total oocytes: T3 vs T1 dietary adherence (Jahangirifar *et al.*, 2019).
DII	1	–	–	–	NS (Diba-Bagtash *et al.*, 2021).	NS: quantity of retrieved and fertilized oocytes. NS: embryo transfer (Diba-Bagtash *et al.*, 2021).

aHEI-2010, alternative healthy eating index 2010; aMED, alternative Mediterranean diet; CHO, carbohydrates; DHA, docosahexaenoic acid; DII, dietary inflammatory index; EPA, Eicosapentaenoic acid; FAs, fatty acids; GI/GL, glycemic index/glycemic load; MedDiet, Mediterranean diet; NS, not significant; −ve/+ve, negative/positive; SSB, sugar sweetened beverages.

#### Carbohydrates and glycemic index/load

Three prospective cohort studies examined glycaemic index, glycaemic load, and carbohydrate intake, two of which were from large population studies such as the ‘Nurses’ Health Study II’ (NH-II) (Chavarro *et al.*, 2009), which included the ‘Snart Foraeldre’ (SF) cohort from Denmark and the ‘Pregnancy Study Online’ (PRESTO) cohort from North America (Willis *et al.*, 2020). The NH-II cohort reported that a higher intake of carbohydrates (highest vs lowest quintile as % of calories, relative risk (RR) (95% CI): 1.91 (1.27–3.02)), and glycemic load (highest vs lowest quintile, RR (95% CI): 1.92 (1.26, 2.92)), was associated with increased risk of ovulatory infertility in adjusted analyses (Table 1). Glycemic index was associated with increased risk of ovulatory infertility, but only in nulliparous women (highest vs lowest quintile, RR (95% CI): 1.55 (1.02, 2.37)). The SF and PRESTO cohorts reported that increasing glycaemic load and total carbohydrates were associated with reduced fecundity (Willis *et al.*, 2020) (Table 1). Whilst dietary fibre intake alone was not found to be associated with fecundity, increasing the carbohydrate–fibre ratio (>13 vs <8, fecundability ratio (FR) (95% CI): 0.86 (0.73, 1.01)) was associated with reduced fecundity. In women undergoing IVF, there was no association between dietary carbohydrate intake and glycemic load with clinical pregnancy rates (Table 2).

#### Fats and fatty acids

Five studies assessed total fat and fatty acids on fertility outcomes. Higher total fat consumption was not associated with ovulatory infertility (Chavarro *et al.*, 2007) or self-reported infertility (Revonta *et al.*, 2010) (Table 1). However, consumption of specific fatty acids had varying and inconsistent effects on fertility outcomes. Higher TFA intakes (each 2% increase in TFA, RR (95% CI): 1.73 (1.09, 2.73)) and replacement of n-6 PUFAs as a proportion of energy with TFA (each 2% energy TFA instead of n-6 PUFAs, RR: (95% CI): 2.31 (1.09, 4.87)) was associated with increased risk of ovulatory infertility (Chavarro *et al.*, 2007). This was incongruous with a different study that reported infertile women aged <50 years had lower intakes of saturated fat than fertile women (OR (95% CI): 0.83 (0.74, 0.92)) (Revonta *et al.*, 2010).

Similar inconsistent results were reported for fecundity. In the SF and PRESTO cohorts, fecundity was lower in women who were in the highest level of TFA intake compared to the lowest level in the PRESTO (Q4 vs Q1, FR: 0.86; 0.71, 1.04), but not the SF cohort (Wise *et al.*, 2018). Similarly, higher saturated fat intake was related to lower fecundity in the North American (Q4 vs Q1, FR: 0.78; 0.72, 0.99) but not Danish cohort (Wise *et al.*, 2018). Higher omega-3 was associated with improved fecundity (Q4 vs Q1, FR: 1.21; 1.01, 1.46) (Wise *et al.*, 2020) (Table 1). In the only included study that investigated fats and fatty acids in women conceiving via ART, the multi-variable adjusted probabilities of live birth for the top quartile of DHA + EPA fatty acid consumption was higher at 54% (95% CI: 42, 66%) compared to the bottom quartile at 36% (95% CI: 26, 48%), but there was no observed effect of total omega-3 consumption. There was also no effect of total or any fatty acids with rates of clinical pregnancy or implantation (Salas-Huetos *et al.*, 2022) (Table 2).

### Core food groups

#### Dairy

Overall, the findings from three studies relating dairy intake with fertility outcomes were inconsistent. In a prospective cohort study, by Afeiche *et al.* (2016), a positive relationship between total dairy intake and live birth rates was reported in women aged >35 years (3 servings/day vs 1.34 servings/day, 21% increase) but not in younger women (Table 1). Wise *et al.* (2017) reported that increased total dairy intake was associated with improved FRs in both Danish and North American cohorts (>18 servings/day vs <7 servings/week, FR (95% CI): 1.37 (1.05, 1.78) in SF and 1.11 (0.94, 1.31) in PRESTO) (Table 1). Chavarro *et al.* (2007) reported no significant association between total dairy intake with ovulatory infertility, after adjusting for energy intake and BMI (Table 1).

The study by Chavarro *et al.* (2007) further stratified dairy consumption by high-fat (whole milk, cream, ice cream, cream cheese, and other cheeses) and low-fat (skim/low-fat milk, sherbet, yogurt, and cottage cheese) dairy formulations, and reported that high fat dairy intake was associated with a reduced risk of ovulatory infertility (high-fat dairy >1 serving/day vs <1 serving/week, RR (95% CI): 0.73 (0.52, 1.01)) (Table 1). Similarly, Wise *et al.* (2017) stratified the analysis by high (summing servings of whole milk, evaporated and condensed milk, whole-milk yoghurt, cheese, ice cream, and mixed recipes) and low-fat formulations (summed servings of skim, reduced-fat chocolate milk, low-fat yoghurt, cottage and ricotta cheese, low-fat cheese, low-fat ice cream, and sorbet) (Table 1). They reported no clear association between low or high-fat dairy content and fecundability in either the North American or Danish cohorts.

#### Whole grains

Two studies assessed the relationship between whole grain intake and fertility, assessed by IVF outcomes (Gaskins *et al.*, 2016; Noli *et al.*, 2020). The study by Gaskins *et al.* (2016) reported a positive association between whole grain intake and live birth rate, with 35% (95% CI: 24, 46) in the lowest quartile of intake (>21.4 g/day) of cycles leading to live birth, compared with 53% (95% CI: 41, 65) in the highest quartile (>52.4 g/day). Furthermore, a 28 g/day increase in whole grain intake was associated with a 0.44-mm (95% CI: 0.1, 0.7) increase in endometrial thickness. However, the second study reported no association between whole grains (regular intake vs no intake) and live birth from IVF (Noli *et al.*, 2020) (Table 2). Both studies reported no association between whole grains and clinical pregnancy (Table 2) (Gaskins *et al.*, 2016; Noli *et al.*, 2020).

#### Fruits and vegetables

Four studies that assessed intake of fruits and/or vegetables in relation to fertility were identified (Revonta *et al.*, 2010; Chiu *et al.* 2018; Grieger *et al.*, 2018; Qu *et al.*, 2019). A multi-centre prospective cohort study by Grieger *et al.* (2018) reported that, compared to women who consumed fruit >3 times/day, consuming fruit 1–3 times/day, 1–6 times/week, or <1–3 times/month, corresponded to a 6% (time ratio (TR) (95% CI): 1.06 (0.97, 1.15)), 11% (TR (95% CI): 1.11 (1.01, 1.22)), and 19% (TR (95% CI): 1.19 (1.03, 1.36)) increase in median time to pregnancy, respectively, as well as a 7% (RR (95% CI): 1.07 (0.88, 1.29)), 18% (RR (95% CI): 1.18 (0.97, 1.44)), and 29% (RR(95% CI): 1.29 (0.95, 1.74)) increased risk of infertility, respectively (Table 1). There was no association with vegetable intake. A cross-sectional study by Revonta *et al.* (2010) reported no association between combined fruit and vegetable consumption and the likelihood of infertility (Table 1). In a prospective nested case–control study, by Qu *et al.* (2019), women who reported a low appetite for vegetables and who were conceiving spontaneously (n = 230 728; 229 917 controls and 811 cases) were twice as likely to experience a stillbirth compared with women reporting a high appetite for vegetables in rural China (OR (95% CI): 1.99 (1.00, 3.93)) (Table 1). The only study that assessed fruit and vegetable intake in women undergoing ART as part of the Environment and Reproductive Health (EARTH) prospective cohort consisting of 325 women reported no effect of total fruit and vegetable intake on live birth, clinical pregnancy, or implantation (Table 2).

#### Fish and seafood

Three studies assessed seafood intake and fecundity in spontaneously conceiving women (Gaskins *et al.*, 2018; Grieger *et al.*, 2018; Wise *et al.*, 2020), while one assessed fish consumption among women requiring ART (Salas-Huetos *et al.*, 2022). Gaskins *et al.* (2018) reported that high seafood consumption was associated with improvements in fecundity (>8 servings/cycle vs <1 servings/cycle, FR (95% CI): 1.60 (1.15, 2.22)), and it was further improved when both the male and female partner were consuming higher intakes of seafood (Table 1). No associations were found between total seafood intake and fecundity in the SF and PRESTO cohorts, however, there was reduced fecundity among women who consumed fried shellfish (>10 g/week vs none, FR (95% CI): 0.77 (0.61, 0.98)) (Table 1). Grieger *et al.* (2018) also reported no association between fish intake and time to pregnancy or infertility in a sample of 5598 women. Salas-Huetos *et al.* (2022) reported that the probability of live birth was higher with the highest quartile of total fish consumption (0.30-1.04 servings/day) (54% (95% CI): (41, 66%)) compared to the lowest quartile (0–0.12 servings/day) (36% (95% CI): (26, 48%)). However, there was no observed effect on rates of clinical pregnancy or implantation.

### Discretionary foods

#### Fast food and take-away food

Two studies reported on fast food and fertility outcomes (Grieger *et al.*, 2018; Lee *et al.*, 2020), of which one defined this as non-home prepared meals (Lee *et al.*, 2020). Among female participants, compared to intakes of fast food ≥4 times/week, the fully adjusted TRs for time to pregnancy were 0.89 (95% CI: 0.81, 0.98) for intake ≥2 to <4 times/week, 0.79 (95% CI: 0.69, 0.89) for intake >0 to <2 times/week, and 0.76 (95% CI: 0.61, 0.95) for no fast food (Grieger *et al.*, 2018). Compared to women consuming fast food ≥4 times/week, the fully adjusted RR of infertility was 0.82 (95% CI: 0.67, 1.00) for women consuming fast food ≥2 to <4times/week, 0.66 (95% CI: 0.51, 0.85) for intake >0 to <2 times/week, and 0.59 (95% CI: 0.37, 0.94) for no fast food. Similarly, Lee *et al.* (2020) reported that women who consumed fast food (>1 meal/day vs none, OR (95% CI): 2.73 (1.15, 6.48)) and non-home prepared meals (>1 meal/day vs none, OR (95% CI): 2.82 (1.48, 5.38)) had higher odds of self-reported infertility (Lee *et al.*, 2020) (Table 1).

#### Soft drinks and sugar-sweetened beverages

Three studies reported on sugar-sweetened beverages in relation to a variety of fertility outcomes (Hatch *et al.*, 2012, 2018; Machtinger *et al.*, 2017). Compared to no intake of softdrinks, softdrink intakes of <1, 1, 2, and >3 servings/day was associated with FRs of 0.89 (95% CI: 0.80, 0.98), 0.85 (95% CI: 0.71, 1.02), 0.84 (95% CI: 0.57, 1.25), and 0.48 (95% CI: 0.21, 1.13), respectively (Hatch *et al.*, 2012). Increased consumption of sugar-sweetened beverages (>7 servings/week vs none, FR (95% CI): 0.81 (0.70, 0.94)) and sugar-sweetened softdrinks (>7 servings/week vs none, FR (95% CI): 0.75 (0.59, 0.95)) was associated with reduced fecundity, but there was no relationship when stratifying by sugar-sweetened fruit juice and energy drinks alone (Hatch *et al.*, 2018). Furthermore, Machtinger *et al.* (2017) reported that women with higher sugar sweetened beverage consumption had, on average, 1.1 fewer oocytes retrieved, 1.2 fewer mature oocytes retrieved, and 0.6 fewer fertilized oocytes compared to women who did not consume sugared softdrink (*P* for trend = 0.002, <0.001, and 0.01, respectively).

### Phytoestrogens

Three studies investigated phytoestrogen intake and fertility outcomes with generally mixed findings (Jacobsen *et al.*, 2014; Vanegas *et al.*, 2015; Wesselink *et al.*, 2020). A large parallel web-based preconception cohort in spontaneously conceiving women from North America (n = 4880) and Denmark (n = 2898) reported no association between soy isoflavone intake and fecundability (Wesselink *et al.*, 2020). However, in a sample of North American Adventist women, Jacobsen *et al.* (2014) reported that after adjustment for age, marital status, and educational status, there was an inverse relationship between soy isoflavone intake and ever becoming a mother (*P* = 0.05). Specifically, soy isoflavone intake was associated with a reduced probability of lifetime live birth by 3% (>40 mg/day vs <10 mg/day (95% CI): 0, 7) and an increased risk of nulligravidity (>40 mg/day vs <10 mg/day, RR (95% CI): 1.13 (1.02, 1.26)). Conversely, dietary soy intake was associated with greater odds of live birth (7.56–27.89 mg/day vs none, OR (95% CI): 1.77 (1.03, 3.03)) (Vanegas *et al.*, 2015) (Table 2).

### Whole diets

Ten studies included holistic dietary approaches, such as the ‘fertility dietary’ pattern, ‘alternate healthy index 2010’ (aHEI-2010), Mediterranean (MedDiet), and alternative Mediterranean diet (aMedDiet), ‘healthy diet’, ‘fertility diet’, ‘pro-fertility diet’, and the dietary inflammatory index (DII).

Higher adherence to the fertility diet, which consisted of higher intakes of vegetable protein, high-fat dairy, monounsaturated fatty acids (MUFA), and iron, with limited intake of low-fat dairy and animal protein, was associated with reduced risk of ovulatory infertility (highest vs lowest adherence, RR (95% CI) 0.34 (0.23, 0.48)) (Chavarro *et al.*, 2007), but no effect on live birth and risk of early pregnancy loss (Gaskins *et al.*, 2014, 2019).

Two studies assessed the aHEI-2010 diet, which constitutes increased intake of fruit and vegetables, nuts, legumes, soy, whole grains, fish and seafood, while limiting intake of red and processed meat, was not associated with early pregnancy loss, miscarriage, stillbirth (Gaskins *et al.*, 2014), or live birth following ART (Gaskins *et al.*, 2019) (Table 2).

One study investigated a ‘healthy dietary’ pattern in an Iranian cohort of 217 infertile women, and reported that higher dietary adherence to a ‘healthy diet’ was associated with improvements in available embryos for implantation (T3 vs T1, *P*-trend = 0.009), while adherence to the ‘unhealthy diet’ had lower odds of clinical pregnancy (T2 vs T1, OR (95% CI): 0.14 (0.3, 0.7)) (Jahangirifar *et al.*, 2019) (Table 2).

The ‘pro-fertility’ diet (developed based on previous factors related to ART outcomes) was linearly associated with ART outcomes, where higher adherence resulted in 47% (95% CI: 21, 77), 43% (95% CI: 19, 72), and 53% (95% CI: 26, 85) higher implantation, clinical pregnancy, and live birth rates, respectively, per SD increase in adherence (Gaskins *et al.*, 2019) (Table 2).

There were seven studies that assessed the MedDiet/aMedDiet with spontaneous and ART fertility outcomes. In an IVF cohort, women who were in the highest quartile of dietary adherence had a higher percentage of live births compared to women in the second quartile (44% (39, 49 vs 31% (24, 39) (Gaskins *et al.*, 2019). There were 8.40 ± 5.26 viable embryos in those who had higher MedDiet adherence compared to 7.40 ± 4.71 in the lower adherence (*P* = 0.028) (Sun *et al.*, 2019). Higher adherence to the MedDiet also reduced the risk of difficulties getting pregnant (highest vs lowest, OR: 0.56; 0.35, 0.95) (Toledo *et al.*, 2011). There were also reports of greater odds of clinical pregnancy (higher adherence, OR: 1.5; 95% CI: 1.0, 1.9) with the MedDiet (Vujkovic *et al.*, 2010) (Table 2). Moreover, a prospective cohort study of non-obese women attending a fertility clinic reported that women in the highest tertile of MedDiet adherence displayed higher rates of clinical pregnancy (29.1 vs 50%, *P* = 0.01) and live birth (26.6 vs 48.8, *P* = 0.01), with no apparent effect on implantation rates (Karayiannis *et al.*, 2018). Another study reported no relationship with MedDiet adherence and several IVF outcomes (good-quality embryos, embryo transfer, as well as others) in analyses adjusted for age, previous ART cycles, and reasons for infertility (Ricci *et al.*, 2019). However, there was a modest reduction in risk of not achieving clinical pregnancy in women >35 years old in the intermediate MedDiet score compared with a low MedDiet score (adjusted RR: 0.84; 95% CI: 0.71, 1.01) (Ricci *et al.*, 2019). Gaskins *et al.* (2014) reported no association between the MedDiet and risk of early pregnancy loss, miscarriage, and stillbirth when adjusting for relevant covariates.

There was no relationship between the dietary inflammatory index and IVF outcomes (Diba-Bagtash *et al.*, 2021).

## Discussion

In this comprehensive and most up to date scoping review on female preconception nutrition, we have identified several nutritional components associated with improved fertility outcomes for women both in the general population and those undergoing ART. A lower dietary energy density, reducing the percentage of daily carbohydrate intake, replacing animal with vegetable protein, reducing TFAs, and reducing discretionary foods appear most likely to have a positive impact on fertility outcomes. Consumption of seafood, dairy, and soy demonstrated inconsistent findings across the few studies examining these. Adherence to the ‘profertility diet’, MedDiet, or a ‘healthy diet’ demonstrated benefits in fertility, while the effects of aHEI2010 and fertility diets were unclear.

The most convincing evidence was for the MedDiet, which demonstrated consistent findings for improving clinical pregnancy and several other fertility outcomes in observational data in both spontaneous and ART pregnancies. The MedDiet involves regular consumption of extra virgin olive oil as the primary source of fat, vitamin-rich foods including plant foods, with moderate consumption of seafood and limited intake of red and processed meat and TFAs (Lacatusu *et al.*, 2019). While the exact mechanisms underpinning the positive effects of the MedDiet on fertility are not completely understood, these are thought to occur by alleviating inflammation, an increasingly recognized factor contributing to poor reproductive and fertility outcomes (Weiss *et al.*, 2009). Many individual MedDiet components, as well as their combination in a whole dietary pattern, have been associated with reduced inflammation and improved fertility outcomes (Chiang *et al.*, 2020). These include extra virgin olive oil being associated with reduced inflammation and improved fertility outcomes (Chiang *et al.*, 2020) through actions of oleocanthal, which is structurally analogous to the steroidal anti-inflammatory agent ibuprofen (Parkinson and Keast, 2014). The components also include vitamins E and C from plant foods, which have been shown to decrease markers of inflammation and oxidative stress in conditions such as endometriosis, likely by ameliorating lipid peroxidation and promoting antioxidant and free-scavenging effects (Mier-Cabrera *et al.*, 2008, 2009). The observations herein, in support of the MedDiet, may therefore stem from the consumption of various foods, nutrients, and bioactive non-nutrient plant compounds (including MUFAs, flavonoids, n-3 and n-6 PUFAs, and relatively limited consumption of TFAs and processed red meat) being linked to reducing chronic low-grade inflammation (Calder *et al.*, 2011; Minihane *et al.*, 2015; Bahr *et al.*, 2021). Furthermore, while healthy dietary approaches are also related to improved weight management and subsequently reduced inflammation; research is increasingly demonstrating that these whole dietary approaches improve inflammation and fertility independent of weight change (Kiddy *et al.*, 1992; Gower *et al.*, 2013; Karayiannis *et al.*, 2018).

The aHEI-2010 score is based on foods and nutrients consistent with population based dietary guidelines that have been shown to lower the risk of chronic disease in clinical and epidemiological studies (Chiuve *et al.*, 2012), while the Fertility Diet score is based on dietary constituents associated with a lower risk of ovulatory infertility identified from epidemiological studies (Chavarro *et al.*, 2007). Results from this scoping review demonstrate the current lack of consistent or clear associations between the aHEI-2010, Fertility Diet and fertility outcomes. For example, the aHEI-2010 was not associated with any fertility outcome assessed (live birth, early pregnancy loss, spontaneous abortion, and stillbirth) (Gaskins *et al.*, 2014, 2019). Moreover, whilst the Fertility Diet was associated with a reduced risk of ovulatory infertility (Chavarro *et al.*, 2007), there was no reported associations with early pregnancy loss, spontaneous abortion, and stillbirth (Gaskins *et al.*, 2014), or live birth, clinical pregnancy or implantation among ART populations (Gaskins *et al.*, 2019). This was unexpected, considering that many of the components of the Fertility Diet, including multivitamins (Czeizel *et al.*, 1996), vegetable protein (Chavarro *et al.*, 2008), and the amount and quality of carbohydrates, have been observed to improve fertility when consumed on their own (Douglas *et al.*, 2006; Shishehgar *et al.*, 2016). Since this food pattern favours low-glycemic foods while limiting intake of TFAs, potential mechanisms could include improved glucose homeostasis and insulin sensitivity, which are crucial for ovulatory function and fertility (Chavarro *et al.*, 2007). Many of the null or inconsistent findings for the Fertility Diet and aHEI-2010 may be due to misclassification bias, when a participant is incorrectly assigned, altering the observed association or research outcome, and lack of independent validation (Chavarro *et al.*, 2007; Gaskins *et al.*, 2014). It is also possible that additional anti-inflammatory components, for example those present in a Mediterranean diet, are required to achieve clinical benefits in addition to the components of a healthy diet; however, this hypothesis awaits further study.

Discretionary foods were consistently reported to be deleterious to fertility outcomes. For example, increased fast food or take aways (specifically, >4 times/week compared to none) was related to increased time to conception and clinical infertility (Grieger *et al.*, 2018), and more than one fast food meal per day was associated with increased self-reported infertility (Lee *et al.*, 2020). Similarly, higher intake of sugar-sweetened beverages was associated with reduced fecundity in spontaneous pregnancy populations, as well as reduced live birth (Hatch *et al.*, 2012, 2018), and ART-specific outcomes such as number of mature and fertilized oocytes in ART populations (Machtinger *et al.*, 2017). This is all consistent with discretionary foods including fast-food, take aways, and sugar-sweetened beverages being associated with increased inflammation through being calorically dense and high in sugar, salt, and SFAs and TFAs (Fuhrman, 2018). Moreover, discretionary foods are also associated with excess weight and women who are overweight or obese have a higher incidence of menstrual dysfunction, anovulation, subfecundity, and infertility (Dağ and Dilbaz, 2015). High-fructose corn syrup is used in sugar-sweetened beverages, which have been reported to contribute ∼6.1–6.9% of daily caloric consumption in the USA (Rosinger *et al.*, 2017). Excess sugar consumption, specifically fructose, directly precipitates cardiovascular disease by dysregulation of lipid, carbohydrate (Cox *et al.*, 2012; Stanhope *et al.*, 2015), and insulin (Meyers *et al.*, 2017) homeostasis independent of excess weight. High-fructose corn syrup intake also has been shown to perturb reproductive organs and increase lipid accumulation in adult female rats (Ko *et al.*, 2017). Hence, discretionary foods, including sugar-sweetened beverages, may have both a direct effect on infertility as well as an indirect effect through obesity with multi-faceted and complex mechanisms including increases in insulin resistance and suboptimal lipid and carbohydrate metabolism (Dağ and Dilbaz, 2015). The potential deleterious effects of higher energy density on some fertility outcomes (e.g. clinical pregnancy, fecundity, and live birth) may also partially reflect discretionary food consumption.

Higher carbohydrate intake, both as a percentage of energy, fibre-poor carbohydrates, and glycemic load were generally associated with poor fertility outcomes while the relationship with glycemic index was unclear. For example, higher percentage carbohydrate intake was associated with higher ovulatory infertility risk (60% vs 42% of calories) (Chavarro *et al.*, 2009), while fibre-poor carbohydrates (>13 vs <8 carbohydrate–fiber ratio) and glycemic load (>141 vs <100) (Willis *et al.*, 2020) were associated with reduced fecundity in spontaneously conceiving populations. However, in the only study that assessed carbohydrate intake in ART populations, there was no relationship between percent carbohydrate intake, glycemic index, and glycemic load with all assessed IVF outcomes (live birth, clinical pregnancy, and embryo transfer) (Noli *et al.*, 2020). The acceptable macronutrient distribution range of carbohydrates is 45–65% for a range of countries (Trumbo *et al.*, 2002; Slavin and Carlson, 2014). Given that some potentially adverse outcomes were observed with percent carbohydrate intakes within this range, future studies are needed to better understand the association of both carbohydrate quality and quantity with fertility outcomes.

Replacing animal with vegetable protein was associated with improved fertility outcomes. For example, replacing 5% of energy from animal protein with vegetable protein reduces the risk of ovulatory infertility in women planning spontaneous pregnancies (Chavarro *et al.*, 2008), and introducing more fish by two servings pre-week in replacement of other meat, protein-rich foods, and processed meat, improved the live birth in ART pregnancies (Nassan *et al.*, 2018). While red meat is considered a good source of protein and nutrients such as iron, zinc and vitamin B12, limiting excess consumption, for example to <455 g/week is recommended by the Cancer Council of Australia (Cancer Council, 2022), due to the presence of high TFA content, various environmental contaminants such as antibiotics (Jeong *et al.*, 2010), and potential carcinogenic effects. Increased TFA consumption may disrupt metabolic pathways and oocyte quality (Cekici and Akdevelioglu, 2019) through mechanisms including down regulation of peroxisome proliferator-activated receptor gamma (PPAR-γ) expression (Clark, 2002; Saravanan *et al.*, 2005), increased inflammatory markers such as C-reactive protein, Interleukin-6, and E-selectin (Baer *et al.*, 2004), as well as a reduction in insulin resistance (Lefevre *et al.*, 2005). Investigating appropriate vegetable and fish sources of dietary protein for optimizing fertility is warranted both for general health benefits and potential fertility benefits.

PUFA consumption demonstrated inconsistent results for fertility. For example, whilst every 2% increase in TFA instead of n-6 PUFAs was associated with higher risk for ovulatory infertility (Chavarro *et al.*, 2007), infertile women <50 years old consumed more total PUFAs than fertile women (6.1% of energy vs 5.8% of energy) (Revonta *et al.*, 2010). However, when stratifying by n-3/n-6 PUFAs, higher consumption of n-3, but not n-6, was associated with improved fecundity (Revonta *et al.*, 2010). In the limited studies in ART populations, increased intakes of n-3 and n-6 PUFA were associated with improved fertility outcomes, including live birth, clinical pregnancy, and blastocyst formation (Jungheim *et al.*, 2013; Al-Safi *et al.*, 2016; Chiu *et al.*, 2018a). Furthermore, systematic reviews consistently report that both n-3 and n-6 PUFAs lower inflammatory and lipid biomarkers in patients with diabetes, cardiovascular disease, and immuno-compromised patients (Rangel-Huerta *et al.*, 2012; Yu *et al.*, 2017; Rangel-Huerta and Gil, 2018; Natto *et al.*, 2019). This likely occurs through their role as precursors to anti-inflammatory eicosanoids, which are biologically active downstream mediators of inflammation (Calder, 2010, 2017). Based on current evidence, higher consumption of certain PUFAs such as n-3 or n-6 appears to have plausible benefits for improving fertility with little to no perceived risks (Molendi-Coste *et al.*, 2011). However, there was inconsistency among the studies included in this review.

Intake of dairy, soy, fruits, and vegetables demonstrated inconsistent results. Dairy has been identified as having a neutral to beneficial effect on fertility and inflammation (Nieman *et al.*, 2020) with this variability likely related to factors including diversity in nutritional composition, bioactive compounds such as fat content, and modes of processing (e.g. fermentation). There have been some reports of potential deleterious effects of soy phytoestrogens on reproductive outcomes in non-human mammals (Seppen, 2012). This has not been confirmed in human RCTs where the observed benefits of isoflavones include increased endometrial thickness and clinical pregnancy rates in patients undergoing uterine insemination (Unfer *et al.*, 2004), nor when it is administered alongside clomiphene induction in unexplained fertility patients who timed intercourse (Shahin *et al.*, 2008). There have also been reports that phytoestrogens when supplemented in the luteal phase may improve the implantation and clinical pregnancy rates in women undergoing IVF (Unfer, Casini, Gerli *et al.*, 2004). In spite of these promising findings, we did not observe these reports in this scoping review. The lack of consistent beneficial association of fruits, vegetables, and whole grains with fertility was surprising given the numerous vitamins, minerals, trace minerals, bioactive nutrients, antioxidants, or non-nutrient components present in these foods (Gutteridge and Halliwell, 2000; Blomhoff, 2005), as well as prior reports of whole grains being associated with endometrial thickness on the day of embryo transfer and improved embryo receptivity and implantation (Gaskins *et al.*, 2016). This may reflect the overall limited number of included studies for these dietary exposures.

### Limitations and future directions

This scoping review offers the most comprehensive and up-to-date search that overviews current observational evidence of the relationships between diet and fertility and evidence gaps that must be filled prior to adoption into clinical practice. Limitations identified in this review include heterogeneity in comparators, exposures and confounders, and observational data precluding causation and making it difficult to reach definitive conclusions on any proposed associations. However, we purposely examined observational studies with the intention of guiding and generating hypotheses for future interventional research. This is a key benefit of observational data and one which should not be underestimated. Indeed, prior systematic reviews have highlighted that existing RCTs examining lifestyle and fertility report on general healthy lifestyle changes only (Lan *et al.*, 2017), and that further research exploring the specific types of dietary interventions is needed. It should also be noted that much of the currently available evidence is not suitable for controlled human studies or RCT designs, and, in this context, observational studies offer considerable insights into the potential benefits or harms, or lack thereof, of specific diets and dietary components for fertility.

Another limitation is that the majority of findings identified herein were derived from a small number of studies and further observational data may be required to inform the design and execution of intervention studies. The limited number of observational studies also precluded sub-group analysis in women with different aetiologies of fertility such as polycystic ovary syndrome (PCOS) or endometriosis and prohibits the pooling of data in meta-analysis. While some studies in this review included the proportion of endometriosis and/or PCOS cases in their patient population (Vanegas *et al.*, 2015; Chiu *et al.*, 2018b; Grieger *et al.*, 2018; Karayiannis *et al.*, 2018; Nassan *et al.*, 2018; Ricci *et al.*, 2019; Noli *et al.*, 2020; Willis *et al.*, 2020), most studies did not, or, in some circumstances, specifically excluded women with such inflammatory conditions (Diba-Bagtash *et al.*, 2021; Hartman *et al.*, 2021). Despite these limitations, there is emerging literature on the relationship between diet and PCOS and endometriosis, which may allow for more stratified sub-group analyses in future. While it is important to assess individual dietary changes, foods are not eaten in isolation and there are likely important additive or synergistic effects of different nutrients in a whole diet setting that were not captured in the included studies. This may partly explain the reason for the consistent evidence found in holistic dietary approaches (predominantly, the MedDiet) compared to the nutrient-specific assessments included in this review. Moreover, the way different diets are analysed in a specific study may impact the results. For example, some dietary pattern studies included in this review adopted a data-driven approach (Vujkovic *et al.*, 2010), while others utilized a priori scoring techniques to define dietary adherence (Gaskins *et al.*, 2014, 2019; Karayiannis *et al.*, 2018). Therefore, discrepancies across studies may reflect differences in methodology rather than actual inconsistencies in the effect of the exposure.

The heterogeneity in geographical origin of these studies similarly impacts the interpretation of these results. Different geographic locales have varying environmental factors influencing production and consumption of food (climate, religion, and culture), and therefore impacting the influence of these dietary exposures in certain patient populations. Moreover, language barriers, specifically in observational studies where the diet is self-reported, may result in classification bias. While there was a wide array of geographical locales investigated, most of the studies were conducted in developed countries (The West, Europe, and Scandinavia), precluding appropriate assessment of these dietary exposures in disadvantaged populations.

Importantly, many included outcomes were in line with the core outcomes set for infertility (Duffy *et al.*, 2021), such as live birth rate, clinical pregnancy rate, early pregnancy loss, and time to pregnancy. However, these were inconsistently reported across the various dietary exposures and between spontaneous and ART populations. There was also variation in the definitions used to assess infertility (e.g. some defined reduced fecundity as higher time to pregnancy using TRs, whereas others assessed fecundity using probability regression modelling to assess the per-cycle probability of conception) which increases opportunities to engage in selective outcome reporting (Duffy *et al.*, 2021). Core outcomes such as gestational age at delivery or core safety measures in ART studies such as neonatal mortality or major congenital anomaly were not reported. Therefore, there is a need for further nutrition-based research on fertility to standardize outcomes and assessment.

### Conclusion

Adherence to the MedDiet and reducing TFA and discretionary foods, show potential benefit in improving fertility outcomes for both spontaneous and ART pregnancies. Seafood, dairy and soy demonstrated inconsistent findings across the few included studies. With the limited and heterogeneous available literature, our findings support the need to further explore interventions to focus on women’s nutrition in the preconception period.

## Supplementary Material

dmad018_Supplementary_Data

## Data Availability

No new data were generated or analysed in support of this work. All data are available via published manuscripts cited in this manuscript.
