# Supplementary material for: Assessing the influence of preconception diet on female fertility: a systematic scoping review of observational studies
Source: Hum Reprod Update. 2023 Jul 19;29(6):811–28. doi: 10.1093/humupd/dmad018 (PMC10663051; doi:10.1093/humupd/dmad018)
Supplement: dmad018_Supplementary_Data [file dmad018_supplementary_data.zip › dmad018_Supplementary_Data/HRU-22-0047-R1-SuppFile1.docx]

Supplementary Data File 1: Search strategy for female preconception nutrition in fertility^1^.

1. preconcep$.tw.
2. pre-concep$.tw.
3. prepregnan$.tw.
4. pre-pregnan$.tw.
5. pregestation$.tw.
6. pre-gestation$.tw.
7. periconception$.tw.
8. peri-conception$.tw.
9. peri conception$.tw.
10. interpregnan$.tw.
11. inter-pregnan$.tw.
12. intergestation$.tw.
13. interconcep$.tw.
14. inter-concep$.tw.
15. inter-gestation$.tw.
16. internatal.tw.
17. inter-natal.tw.
18. before pregnan$.tw.
19. pregnancy intention.tw
20. pregnancy plan$.tw
21. reproductive age*.tw
22. exp Preconception Care/
23. exp family planning services/
24. exp perinatal care/
25. exp Infertility, Female/
26. subfertil$.tw.
27. infertil$.tw.
28. fertility.tw.
29. fecund$.tw
30. in vitro fertili*ation.tw
31. intrauterine insemination.tw.
32. artificial insemination.tw
33. ivf.tw
34. icsi.tw
35. iui.tw.
36. ART.tw.
37. Embryo transfer.tw
38. Assisted reproduc$.tw
39. Assisted conce$.tw
40. Artificial reproduct$.tw
41. or/1–40
42. diet$.tw.
43. nutrition$.tw.
44. nutrient$.tw.
45. food$.tw
46. eat$.tw
47. lifestyle.tw
48. life style.tw
49. intake.tw
50. consum$.tw
51. meat.tw
52. poultry.tw
53. chicken.tw
54. fish.tw
55. seafood.tw
56. soy.tw
57. legume$.tw
58. bean$.tw
59. nut$.tw
60. seed$.tw.
61. dairy.tw
62. fruit.tw
63. vegetable.tw
64. cereal.tw
65. grain$.tw
66. core food.tw
67. non-core food.tw
68. discretionary food.tw
69. non-discretionary food.tw
70. sugar-sweetened.tw
71. soft drink$.tw
72. juice.tw
73. processed food$.tw
74. feeding behaviour/
75. exp food/
76. exp diet/
77. exp diet therapy/
78. exp Life style/
79. or/42–78
80. 41 and 79
81. limit 79 to humans

1 Ovid MEDLINE(R) and Epub Ahead of Print, In-Process & Other Non-Indexed Citations, Daily and Versions(R) <1946 to November 3, 2020>
