# Supplementary material for: Assessing the influence of preconception diet on female fertility: a systematic scoping review of observational studies
Source: Hum Reprod Update. 2023 Jul 19;29(6):811–28. doi: 10.1093/humupd/dmad018 (PMC10663051; doi:10.1093/humupd/dmad018)
Supplement: dmad018_Supplementary_Data [file dmad018_supplementary_data.zip › dmad018_Supplementary_Data/HRU-22-0047.R1 Suppl Tables Final EO.docx]

**Supplementary Table S1: Characteristics of included studies according to energy and macronutrients and core food groups**

| **(First author et al. year), country** | **Population (n) and setting** | **Study design** | **Duration of study (months)** | **Exposure/s** | **Method of diet assessment** | **Spontaneous or ART outcome** | **Outcome/s** |
| --- | --- | --- | --- | --- | --- | --- | --- |
| **ENERGY AND MACRONUTRIENTS** | | | | | | | |
| (Hartman et al. 2021), USA | 29-30 y old couples planning first pregnancy in Northeast region of USA between May 2008 and June 2012 (n = 132). | Prospective cohort | ~6 months (6 menstrual cycles) | Dietary ED: mean energy (kcal)/mean total amount (g) consumed | Three 24-h dietary recalls (2 weekdays, 1 weekend day) | Spontaneous | Clinical pregnancy, live birth, Time to pregnancy |
| (Chavarro et al. 2008), USA | Female registered nurses aged 24 – 42 y old; NH-II in 1989 (n = 18,555) | Prospective cohort | NR | Protein intake | 131-item FFQ | Spontaneous | Ovulatory infertility |
| (Nassan et al. 2018), USA | Couples presenting to Massachusetts General Hospital (MGH) Fertility Center, Boston, 2004 (n=351; 598 ART cycles) | Prospective cohort | On-going (started 2004) | Protein-rich foods | 131-item FFQ | ART | Live birth, clinical pregnancy, implantation |
| (Chavarro et al. 2009), USA | Female registered nurses, aged 24 – 42 y old; Nurses’ Health Study II in 1989 (n = 18.,555) | Prospective cohort | NR | Total carbohydrate intake, GL | 131-item FFQ | Spontaneous | Ovulatory infertility |
| (Noli et al. 2020), Italy | Infertile couples referring to fertility unit in Northern Italy and eligible for IVF between September 2014 and December 2016 (Fondazione IRCCS) (n = 494) | Prospective cohort | 27 | GI/GL, carbohydrate intake, intake of bread and pasta | FFQ | ART | Embryo transfer, clinical pregnancy, live birth |
| (Willis et al. 2020), Denmark and USA | Women 18-45 years attempting pregnancy and not using fertility treatment. Includes 2 web-based cohorts: SF (Danish) (n = 2,709) and PRESTO (North American)(n = 4,269). | Parallel analysis of two prospective cohorts | SF 104, PRESTO 80 | GL, carbohydrate-fiber ratio | Self-administered FFQ in SF cohort; National Cancer Institute’s Diet History Questionnaire II in PRESTO cohort. | Spontaneous | Fecundability |
| (Chavarro et al. 2007), USA | Female registered nurses aged 24 – 42 y old NH- II in 1989 (n = 116,671) | Prospective cohort | Prospective | Total fat, cholesterol, FA | 131-item FFQ | Spontaneous | Ovulatory infertility |
| (Revonta et al. 2010), Finland | Couples participating in nationally representative Health 2000 health interview and examination survey (n = 7,021) | Cross-sectional | N/A | Total fat, SFA, PUFAs, MUFAs, | FFQ | Spontaneous | Infertility |
| (Wise et al. 2018), Denmark and USA  (Wise et al. 2020), Denmark and USA | Women 18-45 y old attempting pregnancy and not using fertility treatment. Includes 2 web-based cohorts: SF (Danish) (n = 1,126) and PRESTO (North American) (n = 1,300).  N = 2,709 in SF and n = 4,269 in PRESTO | Parallel analysis of two prospective cohorts | SF 104, PRESTO 80 | Total fat, SFA, PUFAs, MUFAs, TFA, ω-3 fatty acids, ω-6 fatty acids  Marine fatty acids and marine-sourced long-chain fatty acids (EPA, DHA, DPA) | Self-administered FFQ in SF cohort; National Cancer Institute’s Diet History Questionnaire II in PRESTO cohort. | Spontaneous | Fecundability |
| (Salas-Huetos et al. 2022), USA | Couples presenting to the Massachusetts General Hospital for fertility treatment between 2004 and 2020 (n = 229 couples; 410 ART cycles) | Prospective | 156 | ω-3 intake, and marine-source long-chain fatty acids (DHA, EPA) | 131-item FFQ | ART | Implantation, clinical pregnancy, live birth |
| **CORE FOOD GROUPS** | | | | | | | |
| (Wise et al. 2017), Denmark and USA | Women 18-45 y old attempting pregnancy and not using fertility treatment. Includes 2 web-based cohorts: Snart Foraelfre SF (Danish) (n = 1,116) and PRESTO (North American) (n = 1,300). | Parallel analysis of two prospective cohorts | SF 104, PRESTO 80 | Total dairy, high-fat dairy, low-fat dairy | Self-administered FFQ in SF cohort; National Cancer Institute’s Diet History Questionnaire II in PRESTO cohort. | Spontaneous | Fecundability |
| (Afeiche et al. 2016), USA | Subfertile couples attending the Massachusetts General Hospital (MGH) fertility centre, the Environment and Reproductive Health (EARTH) study started in 2006 (n = 232; 353 ART cycles) | Prospective cohort | 73 | Dairy | 131-item FFQ | ART | Live birth, clinical pregnancy, implantation |
| (Chavarro et al. 2007), USA | Female registered nurses aged 24 – 42 y old at study inception of NH-II in 1989 (n = 18,555) | Prospective cohort | 96 | Dairy | 131-item FFQ | Spontaneous | Ovulatory infertility |
| (Gaskins et al. 2016), USA | Women who underwent at least 1 IVF cycle between February 2007 and August 2014 (n = 264) | Prospective cohort | Pre-IVF to delivery | Whole grain | FFQ: Diet was assessed before IVF treatment initiation | ART | Live birth, clinical pregnancy, implantation |
| (Noli et al. 2020), Italy | Infertile couples referring to fertility unit in Northern Italy and were eligible for IVF between September 2014 and December 2016 (Fondazione IRCCS) (n = 494). | Prospective cohort | 27 | Whole grains, legumes | FFQ | ART | Embryo transfer, clinical pregnancy, live birth |
| (Grieger et al. 2018), Australia, New Zealand, and UK | Nulliparous women with low risk singleton pregnancies from November 2004 to February 2011 (n = 5628) | Multi-centre prospective cohort study | 75 | Fruits, vegetables | Single item questions of specific foods | Spontaneous | Time to pregnancy, infertility |
| (Chiu et al. 2018), USA | Women who completed a dietary assessment and contributed to the Environment and Reproductive Health (EARTH) study between 2007 and 2016. (n = 325; 541 ART cycles) | Prospective cohort | 108 | Fruits, vegetables | FFQ | ART | Live birth, clinical pregnancy, implantation |
| (Revonta et al. 2010), Finland | Couples participating in nationally representative Health 2000 health interview and examination survey (n = 7,021). | Cross-sectional | N/A | Fruits, vegetables, berries | FFQ | Spontaneous | Infertility |
| (Qu et al. 2019), China | 21-49 y old women planning to deliver a baby within 6 months from 31 Chinese provinces (n = 248,501) | Prospective nested-case-control study | 36 | Vegetables | Standardised questionnaire | Spontaneous | Still birth |
| (Wise et al. 2020), Denmark and USA | Women 18-45 y old attempting pregnancy and not using fertility treatment. Includes 2 web-based cohorts: SF (Danish) (n = 2,709) and PRESTO (North American) (n = 4,269). | Parallel analysis of two prospective cohorts | SF 104, PRESTO 80 | Total seafood, types of seafood | Self-administered FFQ in SF cohort; National Cancer Institute’s Diet History Questionnaire II in PRESTO cohort. | Spontaneous | Fecundability |
| (Gaskins et al. 2018), USA | 501 couples attempting to conceive in two geographic areas (Texas and Michigan) from 2005 to 2009, the LIFE study | Prospective cohort | ≤12 | Seafood | Recall and daily journals of seafood consumption | Spontaneous | Time to pregnancy |
| (Salas-Huetos et al. 2022), USA | Couples presenting to the Massachusetts General Hospital for fertility treatment between 2004 to 2020 (n = 229 couples; 410 ART cycles) | Prospective | 156 | Fish | 131-item FFQ | ART | Implantation, clinical pregnancy, live birth |
| **DISCRETIONARY FOODS** | | | | | | | |
| (Grieger et al. 2018), Australia, New Zealand, and UK | Nulliparous women with low risk singleton pregnancies between November 2004 to February 2011 (n = 5628) | Multi-centre prospective cohort study | 75 | Fast food | Single item questions of specific foods | Spontaneous | Time to pregnancy |
| (Lee et al. 2020), USA | 20-49 y old women who participated in 2013-2014 and 2015-2016 National Health and Nutrition Examination Surveys (n = 2,143) | Cross-sectional | N/A | Non-home prepared meals, fast food | Self-reported questionnaire | Spontaneous | Infertility |
| (Hatch et al. 2012), Denmark | 18–40 y old women planning a pregnancy in Denmark (n = 3,628) | Prospective cohort | 36 | Sugar-sweetened beverages and soda | Self-administered beverage-FFQ | Spontaneous | Fecundability |
| (Hatch et al. 2018), USA | 21–45 y old women from pregnancy planners from USA and Canada in the PRESTO cohort who commenced enrolment in 2013 (n = 3,828) | Prospective cohort | ~12 months (until pregnancy or for up to twelve menstrual cycles) | Sugar-sweetened beverages | Self-administered FFQ | Spontaneous | Fecundability |
| (Machtinger et al. 2017), Israel | Women undergoing IVF from 2014 through 2016 for infertility (n = 340) | Prospective cohort | 24 | Sugar-sweetened beverages | Questionnaire on dietary intake | ART | IVF-specific outcomes (total oocytes, mature oocytes, fertilised oocytes, embryo quality), clinical pregnancy, live birth |
| **PHYTOESTROGENS** | | | | | | | |
| (Wesselink et al. 2020), Denmark and USA | Women 18-45 y old attempting pregnancy and not using fertility treatment. Includes 2 web-based cohorts: SF (Danish) (n = 2,898) and PRESTO (North American) (n = 4,880) | Parallel analysis of two prospective cohorts | SF 104, PRESTO 80 | Phytoestrogen | Self-administered FFQ in SF cohort; National Cancer Institute’s Diet History Questionnaire II in PRESTO cohort. | Spontaneous | Fecundability |
| (Vanegas et al. 2015), USA | Infertile women using IVF from 2006 to the present (n = 315; 520 ART cycles) | Prospective cohort | 100 | Soy food | FFQ | ART | Live birth, clinical pregnancy, implantation |
| (Jacobsen et al. 2014), USA | 30-50 y old North American Adventist women with available data regarding childbearing from 2002 to 2007 (n = 11,688) | Cross-sectional | N/A | Soy isoflavones | Self-administered FFQ | Spontaneous | Live birth |
| **WHOLE DIETS** | | | | | | | |
| (Chavarro et al. 2007), USA | Female registered nurses aged 24 – 42 y old NH- II in 1989 (n = 17,544) | Prospective cohort | 96 | FD pattern score (monounsaturated/trans-fat, vegetable protein, high-fat dairy, iron, multivitamins) | 131-item FFQ | Spontaneous | Ovulatory infertility |
| (Diba-Bagtash et al. 2021), Iran | Women undergoing fresh IVF cycles who were attending a fertility centre (n = 144) | Cross-sectional study | 11 | DII | A 3-day 24-h food record diary (two weekdays and one weekend day) | ART | IVF-specific outcomes (fertilization rate, embryo quality), clinical pregnancy |
| (Gaskins et al. 2014), USA | Female nurses aged 24–44 y NH-II at inception in 1989 (n = 11,072; 15,950 pregnancies) | Prospective cohort | Until their first pregnancy loss or the end of follow-up | aHEI-2010, aMED, FD | 131-item FFQ (assessed every 4 years) | Spontaneous | Risk of pregnancy loss, still birth |
| (Gaskins et al. 2019), USA | Women enrolled in the prospective Environment and Reproductive Health (EARTH) Study who had completed at least 1 ART cycle between 2007-2017 (n = 357; 608 ART cycles) | Prospective cohort | 120 | MedDiet, aHEI-2010, the FD, “pro-fertility” diet. | FFQ | ART | IVF-specific outcomes (total oocyte yield, mature oocytes, fertilised oocytes), clinical pregnancy, live birth, implantation |
| (Jahangirifar et al. 2019), Iran | 20-45 y old women undergoing suppression protocol for IVF/ICSI. (n = 140) | Prospective cohort | 6 | “Healthy diet", "western diet", "unhealthy diet" | 168-item validated FFQ | ART | IVF-specific outcomes (total oocytes, retrieved, good quality embryos), fertilisation rate, clinical pregnancy |
| (Karayiannis et al. 2018), Greece | 22-41 y old non-obese women undergoing a first IVF treatment in an Assisted Conception Unit between November 2013 and September 2016 (n = 244) | Prospective cohort | 34 | MedDiet | FFQ | ART | Embryo transfer, implantation rate, fertilisation rate, clinical pregnancy, live birth |
| (Ricci et al. 2019), Italy | Coupled undergoing IVF in an Italian fertility clinic between August 2015 and January 2016 (n = 474) | Prospective cohort | NR | MedDiet | FFQ | ART | IVF-specific outcomes (number of retrieved good-quality oocytes and embryos, embryo transfer), clinical pregnancy, live birth |
| (Sun et al. 2019), China | Infertile women seeking IVF between September 2016 and December 2017 (n = 167) | Prospective cohort | 15 | MedDiet | FFQ | ART | IVF-specific outcomes (number of oocytes retrieved, number of fertilised oocytes, number of available embryos), fertilisation rate, clinical pregnancy, and implantation |
| (Toledo et al. 2011), Spain | Female university graduates started from December 1999 (n = 2154; 485 cases, 1669 controls) | Nested Case Control | 92 | MedDiet | FFQ | Spontaneous | Infertility |
| (Vujkovic et al. 2010), Netherlands | Subfertile couples undergoing IVF-ICSI between September 2004 and January 2007 (n = 161) | Prospective cohort | 28 | MedDiet | FFQ | ART | Clinical pregnancy |

**Abbreviations: aHEI-2010;** alternative healthy eating index 2010; **aMED**, alternative Mediterranean diet; **ART**, assisted reproductive technology; **DHA**, docosahexaenoic acid; **DII**, dietary inflammatory index; **DPA**, docosapentaenoic acid; **ED**, energy density; **EPA**, eicosapentaenoic acid; **FA**, fatty acid; **FD**, fertility diet; **FFQ,** food-frequency questionnaire; **GI**, glycemic index; **GL**, glycemic load; **ICSI**, intracytoplasmic sperm injection; **IVF**, in vitro fertilisation; **MedDiet**, Mediterranean diet; **MUFA**, monounsaturated fatty acid; **N/A**, not applicable; **NH-II**, Nurses’ Health Study-II; **NR**, not reported; **PRESTO**, Pregnancy study online; **PUFA**, polyunsaturated fatty acid; **SFA**, saturated fatty acid; **SF**, Snart Foraeldre; **TFA**, *trans* fatty acid **TTP**, time to pregnancy.

**Supplementary Table S2: Results of included studies.**

| **Study details** | | | | **Results** |
| --- | --- | --- | --- | --- |
| **ENERGY AND MACRONUTRIENTS** | | | | |
| Energy density: 1 | | | (Hartman et al. 2021) | Categorical: Clinical pregnancy (>1.6 kcal/g vs 1.37-1.6, OR: 0.30; 0.11, 0.81); TTP [>1.60 kcal/g vs 1.37-1.60 (HR: 0.40; 0.21, 0.82) ]; live birth [third vs second ED tertile (OR: 0.43; 0.17, 1.07)]  Continuous: Clinical pregnancy, live birth, TTP: NS on controlling for other covariates |
| Protein: 2 | | | (Chavarro et al. 2008) | Ovulatory infertility [highest vs lowest quintile animal protein, RR 1.39 (1.01 to 1.90, 0.03), vegetable protein RR 0.78 (0.54 to 1.12, 0.07)]  Consuming 5% of energy as vegetable rather than animal protein: >50% lower risk of ovulatory infertility (P = 0.007) |
|  |  |  | Nassan 2018 (Nassan et al. 2018) | Live birth: Increasing quartiles of fish, the multi-variable adjusted probabilities were 34.2% (26.5, 42.9), 38.4% (30.3%, 47.3%), 44.7% (36.3%, 53.4%), 47.7% (38.3%, 57.3%)  Increasing fish by 2 servings/wk were replaced with other meat OR 1.54 (1.14, 2.07); other protein-rich food OR 1.50 (1.13, 1.98); or processed meat OR 1.64 (1.14, 2.35)  ART outcomes (clinical pregnancy, live birth, implantation): Total meat intake, eggs, vegetable sources of protein: NS |
| Carbohydrates and Glycemic load: 3 | | | (Chavarro et al. 2009) | Ovulatory infertility: Highest vs lowest quintile of carbohydrate (% of calories) (RR: 1.91; 1.27-3.02); GI was positively related only in nulliparous women |
|  |  |  | (Noli et al. 2020) | IVF outcomes: CHO, GI/GL: NS on controlling for relevant confounders |
|  |  |  | (Willis et al. 2020) | Fecundability ratio: GL < 100 vs GL>141 RR 0.89 (0.73, 1.08) (SF) ; 0.87 (0.77, 0.98) (PRESTO) Carbohydrate-fiber ratio >13 vs <8 0.86 (0.73, 1.01) (SF); 0.87 (0.78. 0.98) (PRESTO); added sugar 27 vs >72 g/d 0.87 (0.68, 1.10) (SF); 0.86 (0.75, 0.99) (PRESTO)  Fecundity: Fiber: NS |
| Fats and FAs: 5 | | | (Chavarro et al. 2007) | Ovulatory infertility: Each 2% increase in TFA (RR = 1.73; 95% CI 1.09, 2.73); each 2% energy TFA instead of n-6 PUFAs (RR = 2.31; 95% CI; 1.09, 4.87)  Total fat, cholesterol, most types of fatty acids: NS |
|  |  |  | (Revonta et al. 2010) | Fertility: Fertile women <50 y old consumed more PUFAs (OR: 1.23; 95% CI: 1.03, 1.46), less saturated fat (OR: 0.83; 95% CI: 0.74, 0.92).  Infertility: Total fat: NS |
|  |  |  | (Wise et al. 2018) | Fecundability ratio: TFA intake Q4 vs Q1 0.86 (0.71, 1.04) in PRESTO, but this was not found in Danish cohort. SFA intake Q4 vs Q1 0.78 (0.62, 0.99) but not in Danish cohort. Omega-3 Q4 vs Q1 1.21 (1.01, 1.46). |
|  |  |  | (Wise et al. 2020) | Fecundity: Marine-sourced long chain fatty acids or total marine fatty acids: NS |
|  |  |  | (Salas-Huetos et al. 2022) | Live birth: Multi-variable adjusted probabilities for the top quartile of DHA + EPA consumption was higher at 54% (95% CI: 42, 66) compared to the bottom quartile at 36% (95% CI: 26, 48%); total omega 3: NS. Early pregnancy loss: Multi-variable adjusted probabilities for top quartile of DHA + EPA consumption lower at 5 % compared to the bottom quartile at 53%. Clinical pregnancy/implantation: All fats and fatty acids: NS |
| **CORE FOOD GROUPS** | | | | |
| Dairy: 3 | | | (Wise et al. 2017) | Fecundability ratio: Total dairy intake >18 servings/week vs <7 servings/week 1.37 (1.05, 1.78) in SF and 1.11 (0.94, 1.31) in PRESTO. Low and high-fat dairy intake: NS. |
|  |  |  | (Afeiche et al. 2016) | Live birth: Total dairy intake (age and calorie-adjusted) difference 3.0 servings/day vs 1.34 servings/day 21% (P = 0.02) in women >35 years but not among younger women (P = 0.69). This relationship did not differ between full-fat or low-fat dairy items. |
|  |  |  | (Chavarro et al. 2007) | Ovulatory infertility: Low-fat dairy foods >2 servings/day per day vs <1 servings/week RR: 1.85 (1.24, 2.77). High-fat dairy >1 servings/day vs <1 serving/week RR 0.73 (0.52, 1.01). Inverse relationship with dairy fat intake (p = 0.05). |
| Whole grains: 2 | | | (Gaskins et al. 2016) | Live birth**:** Whole grain intake >52.4 g/day vs <21.4 g/day, percentages of cycles leading to live birth was 53% (41, 65) and 35% (24, 46), respectively. Endometrial thickness: 28 g/day increase in whole grain intake 0.4mm (0.1, 0.7) increased thickness. |
|  |  |  | (Noli et al. 2020) | IVF outcomes: NS |
| Fruits and vegetables: 4 | | | (Grieger et al. 2018) | Time to pregnancy (TTP): Fruit >3 times/d. 1-3 times/d, 1-6 times/week, or <1-3 times/month led to 6%, 11%, and 19% reductions in median TTP, respectively. Infertility: As compared, to consuming fruit >3 times/d. 1-3 times/d, 1-6 times/week, or <1-3 times/month led to 7, 18, and 29% increased risk, respectively. Vegetable intake: NS. |
|  |  |  | (Revonta et al. 2010) | Fertility: fruits and vegetables: NS |
|  |  |  | (Qu et al. 2019) | Stillbirth: Low appetite for vegetables vs high appetite OR: 1.99 (1.00, 3.93). |
|  | (Chiu et al. 2018) | | Live birth: Total fruits and vegetables: NS; Clinical pregnancy: Total fruits and vegetables: Implantation: Total fruits and vegetables: NS. |  |
| Fish/Seafood: 4 | | | (Wise et al. 2020) | Fecundability ratio: total seafood intake: NS in both cohorts. >10g/week of fried shellfish vs no consumption 0.77 (0.61,0.98). Unfried shellfish: NS. |
|  |  |  | (Gaskins et al. 2018) | Fecundity: couples with female partners who consumed >8 servings/cycle vs <1 serving/cycle had 60% (15, 122) greater fecundity. |
|  |  |  | (Grieger et al. 2018) | Time to pregnancy: fish: NS. Fertility: fish: NS |
|  |  |  | (Salas-Huetos et al. 2022) | Live birth: Total fish: live birth higher in top quartile of fish (0.30-1.04 servings/d) consumption at 54% (95% CI: 0.41, 0.66) compared to bottom quartile (0 – 0.12 servings/d) at 36% (95% CI: 0.26, 0.48). Clinical pregnancy/implantation rate: NS. |
| **DISCRETIONARY FOODS** | | | | |
| Fast food/take-out: 2 | | | (Grieger et al. 2018) | Time to pregnancy: Compared to fast food >4 times/week, >2-4 times/week, 0-2 times/week, or no fast food was 11% (2, 19), 21% (11, 31), and 24% (5, 39) reductions in median TTP, respectively. Infertility: Compared to fast food >4 times/week, >2-4 times/week, 0-2 times/week, or no fast food displayed RR 0.82 (0.67, 1), RR 0.66 (0.51, 0.85), RR 0.59 (0.37, 0.94). |
|  |  |  | (Lee et al. 2020) | Self-reported infertility: >1 fast food meal/day vs none OR: 2.73 (1.15, 6.48). >1 non-home prepared meal/day vs none OR: 2.82; 95% CI: 1.48, 5.38). |
| Sugar-sweetened beverages: 3 | | | (Hatch et al. 2012) | Fecundability ratio: All sodas <1, 1, 2, and >3 servings/day, vs none 0.89 (0.80, 0.98), 0.85 (0.71, 1.02), 0.84 (0.57, 1.25), 0.48 (0.21, 1.13) respectively. |
|  |  |  | (Hatch et al. 2018) | Fecundability ratio: >7 sugar-sweetened beverages/week vs none 0.81 (0.70, 0.94). >7 sugar-sweetened sodas/week vs none 0.75 (0.59, 0.95). Sugar-sweetened fruit juice and energy drinks: NS. |
|  |  |  | (Machtinger et al. 2017) | Live birth: 0.1-1.0 cups sugared soda vs >1 soda was reduced by 12% and 16%, respectively. ART outcomes: Sugar-sweetened beverages vs none had on average 1.1 fewer oocytes retrieved (P = 0.002), 1.2 fewer mature oocytes retrieved (P <0.001), and 0.6 fewer fertilised oocytes (P=0.01) compared to women who did not consume sugar-sweetened beverages. Diet soda: NS. |
| **PHYTOESTROGENS** | | | | |
| Soy and other: 3 | | | (Wesselink et al. 2020) | Fecundity: Phytoestrogen intake: NS. |
|  |  |  | (Vanegas et al. 2015) | Live birth: 0.54-2.64 mg/day, 2.64-7.55, and 7.56-27.89 soy isoflavones vs none OR: 1.32 (0.76, 2.27), 1.87 (1.12, 3.14), 1.77 (1.03, 3.03), respectively. Clinical pregnancy: Any intake of soy foods vs none (11% higher (41, 52), p = 0.03). Implantation: NS. Fertilisation rate: Any soy isoflavone intake vs none (6 % higher (71, 77), p = 0.004). |
|  |  |  | (Jacobsen et al. 2014) | Live birth: Isoflavone intake >40mg/d the lifetime probability of live birth was reduced by 3% (95% CI: 0, 7) vs <10mg/d. Parity: isoflavone intake: NS. Nulligravidity:  isoflavone intake >40mg/day vs <10mg/day 13% (2, 26) higher risk of never have been pregnant. |
| **WHOLE DIETS** | | | | |
| Fertility diet: 3 | | | (Chavarro et al. 2007) | Ovulatory infertility: Quintile 5 vs Quintile 1 of fertility diet adherence RR: 0.34 (0.23, 0.48), even when stratifying by age, parity, and body weight. |
|  |  |  | (Gaskins et al. 2014) | Early pregnancy loss, spontaneous abortion, and still birth: fertility diet adherence: NS. |
|  |  |  | (Gaskins et al. 2019) | Live birth: NS Clinical pregnancy: NS Implantation: NS |
| MedDiet/aMedDiet: 7 | | | (Gaskins et al. 2014) | Early pregnancy loss, spontaneous abortion, and still birth: aMedDiet adherence: NS. |
|  |  |  | (Gaskins et al. 2019) | Live birth: Q4 vs Q2 of MedDiet adherence had 44% probability (39, 49) and 31% (25, 39), respectively. |
|  |  |  | (Ricci et al. 2019) | IVF outcomes: MedDiet score: NS. Clinical pregnancy: Risk of not achieving clinical pregnancy RR: 0.84 (CI: 0.71, 1.09) in women >35 years old. |
|  |  |  | (Sun et al. 2019) | IVF outcomes: higher Mediterranean dietary adherence vs lowest 8.40 +/- 5.26 viable embryos compared to 7.40 +/- 4.71, p = 0.028.  Clinical pregnancy and implantation rate: NS. |
|  |  |  | (Toledo et al. 2011) | Difficulty getting pregnant: Highest adherence of the MedDiet vs lowest OR: 0.56 (0.35, 0.95). |
|  |  |  | (Vujkovic et al. 2010) | Clinical pregnancy: High adherence to the MedDiet OR: 1.40 (1.0-1.9). Fertilisation rate and embryo quality: NS. |
|  |  |  | (Karayiannis et al. 2018) | Implantation: NS. Clinical pregnancy: Low vs high adherence the RR was 0.35 (0.16-0.78). Live birth: Low vs high adherence the RR was 0.32 (0.14-0.71). |
| Healthy diet: 1 | | | (Jahangirifar et al. 2019) | ART outcomes: T3 vs T1 adherence to “healthy diet”, increase in the average number of total oocytes (Ptrend = 0.009) and metaphase II oocytes (0.006).  Clinical pregnancy: T2 vs T1 of “unhealthy diet” adherence OR: 0.14 (0.3, 0.7). |
| aHEI-2010: 2 | | | (Gaskins et al. 2014) | Early pregnancy loss, spontaneous abortion, and still birth: NS. |
|  |  |  | (Gaskins et al. 2019) | Live birth: NS |
| Profertility diet: 1 | | | (Gaskins et al. 2019) | Implantation: 47% (21, 77) higher odds per SD increase in “profertility diet” adherence. Clinical pregnancy: 43% (19, 72) higher odds per SD increase in “profertility diet” adherence. Live birth: 53% (26, 85) higher odds per SD increase in “profertility diet” adherence. Early pregnancy loss: Inversely associated with “profertility diet” adherence OR: 0.69 (0.53, 0.90) per SD increase. |
| DII: 1 | | | (Diba-Bagtash et al. 2021) | ART outcomes: DII, hs-CRP, and IL-6: NS. |
